# Supplementary material for: Prediction of Poststroke Depression Based on the Outcomes of Machine Learning Algorithms
Source: J Clin Med. 2022 Apr 18;11(8):2264. doi: 10.3390/jcm11082264 (PMC9031547; doi:10.3390/jcm11082264)
Supplement: Supplementary file 1 [file jcm-11-02264-s001.zip › jcm-1609237-supplementary.pdf]

Table S1. Correlation analysis between featured parameters in cognitive and functional tests for control and PSD patients

	CNT1_AA OE	CNT1_VA OE	CNTG_VA CR	CNTG_VA CE	MBI1_Dre	MBI1_Tra	MBI2_T	MBI2_Toi	MBI2_Sta	MBI2_Dre	MBI2_Bla	MBI2_Amb	MBI2_Tra	MBIG_Sta	FIM2_T	FIM2_SeC	FIM2_Tra	FIM2_Loc
CNT1_AA OE	-	0.504 *	0.158	0.150	0.021	0.112	0.108	0.114	0.243	0.040	0.000	0.108	0.086	0.193	0.140	0.098	0.156	0.145
CNT1_VA OE	0.504*	-	0.111	0.097	0.086	0.198	0.380*	0.388*	0.482*	0.256	0.213	0.392*	0.329*	0.398*	0.369*	0.353*	0.312*	0.398*
CNTG_VA CR	0.158	0.111	-	0.992*	0.220	0.220	0.458*	0.556*	0.307	0.521*	0.408*	0.370*	0.433*	0.343*	0.505*	0.565*	0.469*	0.421*
CNTG_VA CE	0.150	0.097	.992*	-	0.257	0.207	0.445*	0.542*	0.300	0.513*	0.393*	0.352*	0.413*	0.338*	0.493*	0.564*	0.457*	0.400*
MBI1_Dre	0.021	0.086	0.220	0.257	-	0.549*	0.401*	0.394*	0.317*	0.419*	0.257*	0.380*	0.347*	0.252*	0.388*	0.460*	0.425*	0.395*
MBI1_Tra	0.112	0.198	0.220	0.207	0.549*	-	0.655*	0.621*	0.459*	0.557*	0.531*	0.649*	0.636*	0.393*	0.707*	0.641*	0.735*	0.545*
MBI2_T	0.108	0.380*	0.458*	0.445*	0.401*	0.655*	-	0.916*	0.803*	0.863*	0.778*	0.857*	0.934*	0.752*	0.921*	0.916*	0.861*	0.760*
MBI2_Toi	0.114	0.388*	0.556*	0.542*	0.394*	0.621*	0.916*	-	0.640*	0.820*	0.742*	0.745*	0.852*	0.588*	0.854*	0.892*	0.795*	0.668*
MBI2_Sta	0.243	0.482*	0.307	0.300	0.317*	0.459*	0.803*	0.640*	-	0.660*	0.457*	0.737*	0.712*	0.950*	0.730*	0.672*	0.693*	0.704*
MBI2_Dre	0.040	0.256	0.521*	0.513*	0.419*	0.557*	0.863*	0.820*	0.660*	-	0.630*	0.720*	0.807*	0.612*	0.786*	0.861*	0.808*	0.685*
MBI2_Bla	0.000	0.213	0.408*	0.393*	0.257*	0.531*	0.778*	0.742*	0.457*	0.630*	-	0.537*	0.742*	0.432*	0.731*	0.649*	0.690*	0.496*
MBI2_Amb	0.108	0.392*	0.370*	0.352*	0.380*	0.649*	0.857*	0.745*	0.737*	0.720*	0.537*	-	0.796*	0.701*	0.768*	0.732*	0.706*	0.773*
MBI2_Tra	0.086	0.329*	0.433*	0.413*	0.347*	0.636*	0.934*	0.852*	0.712*	0.807*	0.742*	0.796*	-	0.657*	0.879*	0.868*	0.855*	0.765*
MBIG_Sta	0.193	0.398*	0.343*	0.338*	0.252*	0.393*	0.752*	0.588*	0.950*	0.612*	0.432*	0.701*	0.657*	-	0.674*	0.614*	0.647*	0.641*
FIM2_T	0.140	0.369*	0.505*	0.493*	0.388*	0.707*	0.921*	0.854*	0.730*	0.786*	0.731*	0.768*	0.879*	0.674*	-	0.900*	0.865*	0.765*
FIM2_SeC	0.098	0.353*	0.565*	0.564*	0.460*	0.641*	0.916*	0.892*	0.672*	0.861*	0.649*	0.732*	0.868*	0.614*	0.900*	-	0.861*	0.718*
FIM2_Tra	0.156	0.312*	0.469*	0.457*	0.425*	0.735*	0.861*	0.795*	0.693*	0.808*	0.690*	0.706*	0.855*	0.647*	0.865*	0.861*	-	0.668*
FIM2_Loc	0.145	0.398*	0.421*	0.400*	0.395*	0.545*	0.760*	0.668*	0.704*	0.685*	0.496*	0.773*	0.765*	0.641*	0.765*	0.718*	0.668*	-

Note: Values are Spearman's correlation coefficients. *$p < 0.05$.

Abbreviations: CNT1_AA OE = initial subscore of auditory attention omission error on the CNT; CNT1_VA OE = initial subscore of visual attention omission error on the CNT; CNTG_VA CR = gain subscore of visual attention correct response on the CNT; CNTG_VA CE = gain subscore of visual attention commission error on the CNT; MBI1_Dre = initial subscore of dressing on the K-MBI; MBI1_Tra = initial subscore of transfer on the K-MBI; MBI2_T = follow-up total score on the K-MBI; MBI2_Toi = follow-up subscore of toileting on the K-MBI; MBI2_Sta = follow-up subscore of stair-climbing on the K-MBI; MBI2_Dre = Follow-up subscore of dressing on the K-MBI; MBI2_Bla = follow-up subscore of bladder control on the K-MBI; MBI2_Amb = follow-up subscore of ambulation on the K-MBI; MBI2_Tra = follow-up subscore of transfer on the K-MBI; MBIG_Sta = gain subscore of stair-climbing on the K-MBI; FIM2_T = follow-up total score on the FIM; FIM2_SeC = follow-up subscore of self-care on the FIM; FIM2_Tra = follow-up subscore of transfer on the FIM; FIM2_Loc = follow-up subscore of locomotion on the FIM

Table S2. Correlation analysis between featured parameters in cognitive and functional tests for PSD patients

	Education period	MMSE2_T	MMSE2_Rec	MMSEG_Rec	CNT2_LM DSF	CNT2_LS DSB
Education period	-	0.127	0.200	0.218	0.212	0.304
MMSE2_T	0.127	-	0.772 *	0.420 *	0.736 *	0.766 *
MMSE2_Rec	0.200	0.772 *	-	0.825 *	0.856 *	0.867 *
MMSEG_Rec	0.218	0.420 *	0.825 *	-	0.721 *	0.646 *
CNT2_LM DSF	0.212	0.736 *	0.856 *	0.721 *	-	0.859 *
CNT2_LS DSB	0.304	0.766 *	0.867 *	0.646 *	0.859 *	-

Note: Values are Spearman's correlation coefficients. *$p < 0.05$.

Abbreviations: MMSE2_T = follow-up total score on the K-MMSE; MMSE2_Reg = follow-up subscore of registration on the K-MMSE; MMSEG_Rec = gain subscore of recall on the K-MMSE; CNT2_LM DSF = follow-up subscore of digit span forward language memory on the CNT; CNT2_LM DSB = follow-up subscore of DSB language memory on the CNT

Table S3. Accuracy analysis using various hyper-parameters for the prediction of PSD occurrence and prognosis with 5-fold cross-validation (A), and 10-fold cross-validation (B)

(A)

	Control vs. PSD				Imp vs. NoImp			
Kernel	SVM_L		SVM_R		SVM_L		SVM_R	
gamma<br>C	scale	auto	scale	auto	scale	auto	scale	auto
1	0.600	0.600	0.569	0.569	0.771	0.771	0.648	0.648
2	0.600	0.600	0.585	0.585	0.738	0.738	0.614	0.614
3	0.569	0.569	0.631	0.585	0.771	0.771	0.614	0.614
4	0.600	0.600	0.646	0.600	0.771	0.771	0.614	0.648
5	0.600	0.600	0.631	0.646	0.771	0.771	0.648	0.648
6	0.569	0.569	0.646	0.600	0.771	0.771	0.648	0.648
7	0.569	0.569	0.646	0.646	0.771	0.771	0.648	0.648
8	0.569	0.569	0.631	0.585	0.771	0.771	0.648	0.648
9	0.585	0.585	0.646	0.600	0.771	0.771	0.614	0.614
10	0.600	0.600	0.631	0.646	0.771	0.771	0.614	0.614

(B)

	Control vs. PSD				Imp vs. NoImp			
Kernel	SVM_L		SVM_R		SVM_L		SVM_R	
gamma<br>C	scale	auto	scale	auto	scale	auto	scale	auto
1	0.636	0.636	0.619	0.619	0.775	0.775	0.675	0.675
2	0.636	0.636	0.593	0.593	0.775	0.775	0.642	0.642
3	0.636	0.636	0.626	0.638	0.775	0.775	0.642	0.642
4	0.636	0.636	0.669	0.655	0.775	0.775	0.642	0.642
5	0.636	0.636	0.655	0.655	0.775	0.775	0.642	0.642
6	0.636	0.636	0.700	0.700	0.775	0.775	0.675	0.642
7	0.636	0.636	0.698	0.683	0.775	0.775	0.675	0.642
8	0.619	0.619	0.683	0.683	0.775	0.775	0.708	0.708
9	0.619	0.619	0.683	0.683	0.750	0.750	0.708	0.708
10	0.619	0.619	0.683	0.683	0.750	0.750	0.708	0.708

Abbreviations: PSD = poststroke depression; Imp = PSD patients showing improvement in their symptoms; NoImp = PSD patients showing no symptom improvement; SVM_L = linear support vector machine; SVM_R = support vector machine with radial basis function kernel function, C = Regularization parameter In SVM, gamma = kernel coefficient.
